# Translation, adaptation, and pilot of a guided self-help intervention to reduce psychological distress in South Sudanese refugees in Uganda

**DOI:** 10.1017/gmh.2018.14

**Published:** 2018-07-27

**Authors:** W. A. Tol, J. Augustinavicius, K. Carswell, F. L. Brown, A. Adaku, M. R. Leku, C. García-Moreno, P. Ventevogel, R. G. White, M. van Ommeren

**Affiliations:** 1 PCAF Global Mental Health Program, Health Right International, Kampala, Uganda; 2 Department of Mental Health, Johns Hopkins Bloomberg School of Public Health, Baltimore, MD, USA; 3 Department of Mental Health and Substance Abuse, World Health Organization, Geneva, Switzerland; 4 WarChild Holland, Amsterdam, the Netherlands; 5 Department of Global Health and Population, Research Program for Children and Global Adversity, Harvard T. H. Chan School of Public Health, Boston, MA, USA; 6 Arua Regional Referral Hospital, Arua, Uganda; 7 Department of Reproductive Health & Research, World Health Organization, Geneva, Switzerland; 8 Public Health Section, United Nations High Commissioner for Refugees, Geneva, Switzerland; 9 Institute of Psychology Health and Society, University of Liverpool, Liverpool, UK

**Keywords:** Adaptation, interventions, pilot study, psychological distress, refugees, self-help

## Abstract

**Background.:**

In this period of unprecedented levels of displacement, scalable interventions are needed to address mental health concerns of forced migrants in low-resource settings. This paper describes the adaptation and piloting of a guided, multi-media, self-help intervention, Self-Help Plus (SH+), which was developed to reduce psychological distress in large groups of people affected by adversity.

**Methods.:**

Using a phased approach that included community consultations, cognitive interviewing, facilitator training, pilot implementation, and a qualitative process evaluation, we adapted SH+ for use among South Sudanese refugees in a refugee settlement in northern Uganda.

**Results.:**

The SH+ materials, including audio-recorded sessions and an accompanying illustrated manual, were translated into Juba Arabic. Cognitive interviewing primarily resulted in adaptations to language with some minor adaptations to content. Facilitator training and supervision led to further suggested changes to delivery methods. An uncontrolled pilot study (*n* = 65) identified changes in the expected direction on measures of psychological distress, functional impairment, depression, wellbeing, and psychological flexibility. The process evaluation resulted in further adaptations to intervention materials and the decision to focus future effectiveness evaluations of the intervention in its current form on South Sudanese female refugees.

**Conclusions.:**

We found that this potentially scalable, guided self-help intervention could be adapted for and feasibly implemented among female South Sudanese refugees in northern Uganda. These findings lay the groundwork for a future rigorous evaluation of SH+ in this context.

## Introduction

The world is currently experiencing unprecedented levels of forced migration. A total of 22.5 million refugees, 40.3 million internally displaced people, and 2.8 million asylum seekers were documented in 2016 (UNHCR, 2017a). Most forced migrants live in low- and middle-income countries (LMIC) (UNHCR, 2017a), where the majority of the world's armed conflicts take place (Melander *et al.*, 2016). A recent systematic review and epidemiological modeling study with conflict-affected populations in non-Western settings found age-standardized pooled prevalence rates of 12.9% for post-traumatic stress disorder (PTSD) (66 studies, 27 countries) and 7.6% for depression (33 studies, 20 countries) (Charlson *et al.*, 2016). In keeping with this epidemiological literature, intervention research in LMIC humanitarian settings has often focused on evaluating psychotherapeutic interventions for PTSD and depression (Tol *et al.*, 2011).

There are multiple barriers that can limit the ability of current evidence-based interventions to improve population mental health in humanitarian settings. First, many people in humanitarian settings suffer from broad psychological distress, for example, non-pathological anxiety, grief reactions, and demoralization, that cannot be easily categorized as mental disorder (WHO & UNHCR, 2012; Tol *et al.*, 2013b). Stress management strategies may be a more appropriate approach for this population. Second, although there is evidence from randomized controlled trials supporting psychotherapies (e.g., for PTSD, trauma-focused cognitive behavioral therapies and eye movement desensitization and reprocessing) (Tol *et al.*, 2013a) such interventions have limitations with regard to their feasibility for scale-up in disrupted and under-resourced health systems, given the need for an extensive workforce of adequately trained providers and supervisors (Tol *et al.*, 2014; Silove *et al.*, 2017). Third, current evidence-based psychotherapeutic treatments have been conventionally designed to target single mental disorders, whereas comorbidity of common mental disorders is high in populations affected by armed conflict (de Jong *et al.*, 2003). Training workers in multiple psychotherapies would require significant resources. Fourth, the application of psychotherapies evaluated in well-resourced randomized controlled trials to real-world practice settings represents a widely-recognized challenge (Weisz *et al.*, 2005) and less resource-intensive interventions may be more successful in bridging this gap (Bennett-Levy *et al.*, 2010).

As part of efforts to make available a series of scalable psychological interventions, the World Health Organization (WHO) has developed a stress management package based on a guided self-help format called Self-Help Plus (SH+) that may address aforementioned barriers (Epping-Jordan *et al.*, 2016). A guided, multi-media, self-help format was selected informed by various meta-analyses: (1) research has shown promising findings for bibliotherapy (den Boer *et al.*, 2004) and group psychosocial interventions formatted as courses (Cuijpers *et al.*, 2009); (2) guided self-help approaches have similar benefits to face-to-face psychotherapeutic interventions (Cuijpers *et al.*, 2010; Lewis *et al.*, 2012); and (3) guided self-help interventions are associated with larger effect sizes than unguided self-help interventions (Hirai & Clum, 2006; Cuijpers *et al.*, 2009).

In this paper, we describe the translation, adaptation, and the first uncontrolled piloting of SH+ with South Sudanese refugees in northern Uganda. In a participatory needs assessment in the study area, ‘overthinking’ was the highest ranked mental health and psychosocial concern amongst refugees in Rhino Camp (Adaku *et al.*, 2016). ‘Thinking too much’ is a cultural concept of distress that has been repeatedly identified in a wide range of settings (Kaiser *et al.*, 2015), including in qualitative studies with refugees from southern Sudan before independence (Coker, 2004; Goodman, 2004) and South Sudan (Ventevogel *et al.*, 2013). Given these findings, we anticipated that the focus of SH+ on strategies for managing difficult thoughts and feelings would be perceived as highly relevant. At the same time, we expected that adaptations would be required to enhance acceptability, feasibility, and satisfaction with the intervention in this socio-cultural context.

Several frameworks for the cultural adaptation of psychological interventions have been published (Bernal *et al.*, 1995; Hwang, 2009; Barrera *et al.*, 2013) with adapted interventions appearing to be more effective than non-adapted interventions (Benish *et al.*, 2011; Harper Shehadeh *et al.*, 2016). Adaptation frameworks commonly suggest iterative processes that emphasize engaging with stakeholders through qualitative research approaches before piloting the intervention using process evaluation methods. Adapted interventions may then be tested using controlled designs (Medical Research Council, 2008; Chowdhary *et al.*, 2014). Typically, researchers have taken a ‘middle ground’ approach in which adaptations are made to enhance acceptability and effectiveness while maintaining fidelity to the core elements of the psychological intervention (Chowdhary *et al.*, 2014). WHO is in the process of developing a systematic psychological intervention adaptation framework, inspired, in part, by earlier work on translation and adaptation of instruments (van Ommeren *et al*., 1999). The current study is the first effort to apply key aspects of this framework and adapt a self-help intervention with refugees in a LMIC.

## Methods

### Setting and population

The study was conducted in the Rhino Camp refugee settlement in the West Nile region of northern Uganda, which is inhabited primarily by refugees from South Sudan. South Sudan became an independent nation in 2011 after decades of civil war, however, following an alleged attempted coup in December 2013 conflict resurged, and fighting subsequently re-intensified in July 2016. By September 2017, the conflict had displaced 4.3 million people, including more than 2 million people seeking refuge in the region. Uganda hosts the largest number of South Sudanese refugees in the region (over a million), of whom 223097 currently live in Rhino Camp (UNHCR, 2017b). Sexual and other forms of gender-based violence are one of the significant concerns among South Sudanese refugees, with more than half of women aged 15–24 have experienced at least one form of gender-based violence (Protection Cluster South Sudan - GBV Subcluster, 2015).

### Intervention

SH+ is intended to decrease psychological distress in people with or without a diagnosed condition associated with a range of adversities (e.g., interpersonal and collective violence, chronic poverty), but is not intended for participants with severe health problems such as psychosis or imminent risk of suicide (Epping-Jordan *et al.*, 2016).

The intervention is based on principles of Acceptance and Commitment Therapy (ACT), a third-wave cognitive behavioral therapy that aims to enhance psychological flexibility (Hayes *et al.*, 2006). Psychological flexibility reflects how a person: adapts to fluctuating situational demands; reconfigures mental resources; shifts perspective, and balances competing desires, needs, and life domains (Kashdan & Rottenberg, 2010). Based on the premise that attempts to suppress or avoid unwanted thoughts and feelings may paradoxically worsen them, ACT teaches alternative methods to accommodate difficult thoughts and feelings, primarily through mindfulness techniques. At the same time, ACT also focuses on guiding participants to live in ways consistent with their personal values (Hayes *et al.*, 2011). Studies have suggested that ACT may be effectively applied in a self-help format (Cavanagh *et al.*, 2014) and in diverse cultural contexts (White *et al.*, 2017).

The SH+ package comprises a pre-recorded audio course and an illustrated self-help manual. The audio-course can be delivered to groups of 20–30 people by lay facilitators trained over a short period. The course consists of five weekly 2-hour sessions that include individual exercises and small group discussions. The illustrated manual, inspired by an existing book (Harris, 2014), covers the key points from the course and is provided for participants to review on their own time in between sessions. Initial development of the generic intervention took place in response to the crisis in Syria, with the generic version designed to be applicable, once adapted, to diverse socio-cultural settings.

### Stakeholder involvement

In the early stages of intervention planning, a community stakeholder meeting was held. The meeting was attended by refugee leaders, traditional leaders, religious leaders, respected elders, and school principals. These individuals were invited to join a community advisory board, which was consulted at various stages throughout the project. The project and intervention were also formally presented at an inter-agency meeting within Rhino Camp, which was attended by government stakeholders as well as representatives of non-governmental organizations working in the refugee settlements.

Following situation analysis involving secondary data analysis and qualitative interviews (Adaku *et al*., 2016), adaptation and piloting took place in four phases: (1) translation and cognitive interviewing; (2) adaptation during training and supervision; (3) pilot implementation; and (4) process evaluation.

### Phase 1: Translation and group cognitive interviewing

South Sudan has 68 living languages, with the most widely spoken languages being used mainly within ethnic groups (e.g., Nuer, Dinka, Bari, and Zande) (Lewis *et al.*, 2015). Translation into one of these languages would limit accessibility for other ethnic groups, and may have been perceived as privileging one ethnic group over another. Instead, Juba Arabic is a common lingua franca (UNICEF, 2016), although a reliable figure for the number of Juba Arabic speakers is not available (Lewis *et al.*, 2015). Juba Arabic is spoken by the large majority of refugees and is the language most commonly used by humanitarian agencies in interactions with refugees. As advised by the community advisory board, we translated SH+ materials into Juba Arabic.

A complete translation of intervention materials from English to Juba Arabic was conducted by a bi-lingual speaker with experience working in humanitarian health programs in the region. This translation was subsequently reviewed and edited by a Juba Arabic speaking research assistant. The illustrator of the generic WHO SH+ manual adapted the illustrations so that they would be appropriate for the study context (e.g. changing characters, styles of clothing, housing, environment, and common cultural practices).

We subsequently applied an adapted cognitive interviewing approach. Cognitive interviewing is commonly used to check whether the meaning of survey questions has been translated accurately (Beatty & Willis, 2007), but has also been applied in the development of interventions such as health education materials (Carbone *et al.*, 2002). In general, cognitive interviewing entails presenting participants with text and then asking questions about how the participant interprets the meaning of the text (Beatty & Willis, 2007). Cognitive interviews were facilitated by trained interviewers residing in the region. We were interested in conducting group cognitive interviews with two separate groups of 8–12 people: one group of health workers and one group of laypersons. During group interviews, we read aloud brief segments of the translated script for the audio recordings, showed images from the illustrated manual, and asked questions focused on relevance, comprehensibility, and acceptability (van Ommeren *et al.*, 1999). During these sessions, a semi-structured form was used to record comments applying to comprehensibility, acceptability, relevance, as well as proposed changes pertaining to each segment of script or illustration.

Proposed changes were later transferred to a structured form that included the eight adaptation dimensions as described by Bernal *et al.* (1995) and by Chowdhary *et al*. (2014). An adaptation workshop was held in November 2015 with intervention developers and the research team to discuss these proposed changes and adapt the intervention materials.

### Phase 2: Adaptations suggested during training and supervision

A need for further adaptations to SH+ was identified during the training of facilitators and by facilitators during the first pilot groups. Training was provided to four female SH+ facilitators (selected to have completed secondary education; proficient spoken Juba Arabic; and good English language skills) over 5 days by two authors (KC, FB) involved in the development of SH+. All four facilitators were Ugandans living in the settlement region with previous experience working in the settlement. Changes to SH+ were identified during the training, recorded in field notes and later categorized using the Bernal *et al*. (1995) framework.

Regular (mostly weekly) supervision calls were completed with one author (KC) and the social worker supervisor was available for emergency cases and responding to serious additional needs of participants (e.g. reported risk issues). Further changes were identified during the regular supervision. Where possible, agreed adaptations were incorporated before the pilot implementation. Larger changes to content were considered after the pilot implementation, due to the time required to edit pre-recorded audio content.

### Phase 3: Pilot implementation

#### Objective and overall design

The objective of the pilot study was to assess feasibility and acceptability of the intervention and research protocols, and this was evaluated quantitatively through an uncontrolled pre-post design followed by a qualitative process evaluation (see Phase 4).

#### Sampling and recruitment

Participants for two SH+ groups were recruited from one village in Rhino Camp. We consulted with the village chief and community health workers about people whom they thought should be screened for eligibility. In addition, we applied a general door-to-door recruitment strategy, with research assistants familiar within the community moving from house to house until sufficient people were enrolled to form one female and one male SH+ group of around 25–30 participants.

#### Procedures

South Sudanese men and women over 18 years of age were informed by the research team about the study and asked if they would be willing to participate in screening. Among those interested in the study, informed consent was obtained and screening measures were administered in Juba Arabic by trained research assistants.

#### Eligibility and measures

Screening focused on psychological distress measured using the Kessler 6 (K6), a questionnaire that assesses non-specific psychological distress (i.e., distress not specifically tied to particular mental disorders) in the last 30 days through six questions with a 0 (not at all) to 4 (all the time) response format (total score range 0–24) (Kessler *et al.*, 2003). A person who scored five or above (moderate psychological distress) on the K6 was considered eligible for inclusion in the study (Prochaska *et al.*, 2012). Exclusion criteria included: (1) imminent risk of suicide (assessed using a structured protocol); (2) observable signs of psychosis, manic behaviors, or intellectual disability that impede participation in a guided self-help intervention (assessed through observation using a structured checklist). Participants excluded for these reasons were referred to a mental health professional.

Among participants who met inclusion criteria, we assessed violence exposure (the WHO Violence Against Women Instrument, WHO-VAWI) (Garcia-Moreno *et al.*, 2006); functional impairment (World Health Organization Disability Assessment Schedule 2.0, 12-item interviewer-administered version, WHODAS 2.0) (Ustun *et al.*, 2010); depression (the Patient Health Questionnaire, PHQ-9) (Kroenke *et al.*, 2001); subjective psychological wellbeing (5-item WHO Well-Being Index, WHO-5) (Topp *et al.*, 2015); and psychological flexibility (Acceptance and Action Questionnaire-II, AAQ-II) (Fledderus *et al.*, 2012). The same measures, including the K6, were re-administered within 2 weeks post-intervention.

#### Intervention implementation

The day before each SH+ session, intervention facilitators visited participants’ homes to remind them of the upcoming group. Groups were conducted with two facilitators in each group (one to control the audio and the other to respond to participants), with facilitators completing a session reporting form after each group.

#### Analyses

We used descriptive statistics to describe socio-demographic characteristics of the sample at baseline. Total scale scores were calculated for each participant and these scale scores were averaged. To assess psychometric properties of measures, we calculated internal consistency (Cronbach's Alpha) and we examined bi-variate Pearson correlations between the average sum of scores on scales to assess convergent and divergent validity. After assessing the assumptions of the paired samples *t* test, pre- and post-assessment measures were compared using paired samples *t* tests or the Wilcoxon signed-rank test to examine sensitivity to change and to analyse the general direction of changes before and after intervention. Statistical analyses were carried out using Stata 13 (Statacorp, 2013).

### Phase 4: Process evaluation

The process evaluation consisted of semi-structured interviews with: participants who completed all five sessions (*N* = 8); participants who discontinued the intervention (*N* = 10); SH+ facilitators (*N* = 4); and one supervising social worker. Interviews with intervention participants were conducted in Juba Arabic, and with other key informants in English. Interviews were recorded, transcribed, and Juba Arabic interviews were then translated into English. Participants who attended all sessions were asked about their overall impressions of the program; attendance; involvement of family and friends; helpfulness; appropriateness; and components deemed most useful. Participants who attended sessions intermittently or stopped attending were asked about similar topics, with the exception of components deemed most useful. They were also asked about reasons for missing sessions. In addition to overall impressions, facilitators were asked specifically about challenges in facilitation, language, training and supervision, and the most useful components of the intervention. The social worker was asked about intervention delivery, implementation, scale-up, and potential for integration into primary or community healthcare.

Thematic analysis of key informant interviews involved first reading the full set of transcripts and then developing codes using a subset of transcripts. Once a codebook had been developed, the full set of transcripts were coded, with additional codes added where required. The final set of codes was then examined and at points combined to represent overarching themes within the following categories: benefits; challenges and barriers; and suggestions for the future. Themes were then revised according to their fit with the data. All qualitative analyses were carried out using Atlas.ti (ATLAS.ti Scientific Software Development GmbH, 2016).

### Ethics

All participants signed informed consent forms or noted consent with a thumbprint. Study procedures were approved by the WHO Ethics Review Committee, Johns Hopkins Bloomberg School of Public Health Institutional Review Board, the Lacor Hospital Institutional Research and Ethics Committee, and the Uganda National Council for Science and Technology.

## Results

### Phase 1: Cognitive interviews

Group cognitive interviews were carried out with one group of health workers and one group of laypersons. Cognitive interviews with mental health workers were held over a 2-day meeting. Health workers proposed 31 changes to the illustrated manual and 42 to the audio script. Proposed changes to the illustrated manual included replacing text and adapting illustrations (e.g., adding props for clarity of interpretation, improving the local relevance of images by changing hairstyles or adding locally understood signs). The majority of the proposed changes were related to language rather than to changes in underlying intervention concepts (e.g., exchanging the phrase ‘knots in the stomach’ for ‘discomfort in the stomach’). Language is one of eight dimensions in the Bernal *et al.*, framework, which also includes: persons, metaphors, content, concepts, goals, methods, and context (Bernal *et al.*, 1995). In addition to language, some suggestions were made regarding metaphor and concepts. Moreover, the group cognitive interviews with health workers identified few suggestions for adaptations on the dimensions of persons and goals. These are changes that can be made to the generic WHO intervention so that in future such adaptations would not be necessary.

The group of lay people consisted of 12 South Sudanese refugees of both sexes and various ages. Over six meetings the group participated in a complete read through of the SH+ audio script and reviewed some examples of adapted and non-adapted images from the illustrated manual. The group did not suggest any changes to the audio script and only proposed three changes to images. Overall, participants indicated that each of the sections in the materials covered during cognitive interviewing were comprehensible, relevant, and acceptable. To determine whether the lack of suggested changes reflected accurately how well the materials were comprehensible to participants, additional questions were asked to check understanding of concepts. This yielded mixed responses. For example, some participants adequately described the SH+ concept of ‘grounding’, but others provided generic responses describing the purpose of SH+ in terms of ‘giving counselling to people who need it’. Participants frequently re-iterated that the intervention was seen as helpful, but were not always able to explain specifically how. Participants emphasized the relevance of many of the SH+ concepts to their community due to the stresses that they were facing and did not indicate that any of the text or illustrations were offensive.

### Phase 2: Adaptations suggested during training and supervision

During training, facilitators suggested changes primarily to the delivery method of SH+, including providing a longer spoken introduction, using energizer games or an opening activity consistent with practice in Rhino camp (e.g., singing and dancing), setting ground rules as a group, and providing further explanations of key concepts where required. The initial concept of SH+ was for facilitators to have an exceptionally minimal role, running the group and ensuring safety, but giving little or no feedback or direction. All facilitators suggested that for participants this would be unusual and different from previous experiences, so the facilitator role was adapted to somewhat increase their presence in the process by starting the group, introducing the audio, and giving increased (but still minimal) clarifications and instructions to participants. During training, facilitators reported some content (e.g. a repeated grounding exercise) was too long (though these changes could not be incorporated before the Phase 3 pilot). During and after supervision, facilitators provided additional suggestions for changes separate to the process evaluation including, removal of worksheets (due to literacy concerns), simplifying the audio script, reducing the length of sessions and some exercises, adding audio cues to signal when a facilitator had to do an activity, and showing relevant illustrations from the SH+ manual to support understanding of key concepts. These changes were incorporated after the pilot.

### Phase 3: Pilot implementation

#### Characteristics at baseline

A total of 86 men and women were screened for participation and 65 (76%) (33 women, 32 men) who scored above 5 on the K6 were included in the study (Table 1). Participants on average reported severe mental distress at baseline (*M* = 14.5, s.d. = 4.1), as compared with the commonly used cut-off score of 13 for severe psychological distress on the K6. Participants reported moderate functional impairment on the WHODAS (*M* = 26.7, s.d. = 6.7) and moderate levels of depression on the PHQ-9 (*M* = 12.8, s.d. = 5.2). Wellbeing scores based on the WHO-5 were relatively low in this sample (*M* = 33.6, s.d. = 21.2). With regard to interpersonal violence, physical violence by a partner was reported by 15 women (45%) and five men (16%). Non-partner violence was reported by 11 women (33%) and nine men (28%) (physical violence) and two women (6%) and two men (6%) (sexual violence).

**Table 1.** Demographic characteristics of the pilot sample at pre-assessment

Characteristics	Females (*N* = 33)	Males (*N* = 32)
Age, Mean (s.d.)	35.4 (10.5)	33.5 (13.7)
Years of education, Mean (s.d.)	2.8 (3.6)	7.5 (5.1)
Marital status, No. (%)		
Never married	1 (3)	12 (38)
Currently married	11 (33)	17 (53)
Separated	9 (27)	3 (9)
Widowed	12 (37)	–
Employment, No. (%)		
Paid work	1 (3)	1 (3)
Self-employed	10 (30)	15 (47)
Non-paid work	1 (3)	2 (6)
Student	–	2 (6)
Keeping house/homemaker	20 (61)	3 (10)
Retired	–	2 (6)
Unemployed	–	4 (12)
Other	–	3 (10)
Missing	1 (3)	–
Ever experienced physical violence, No. (%)		
By partner	15 (45)	5 (16)
By non-partner	11 (33)	9 (28)
Sexual violence by non-partner, No. (%)	2 (6)	2 (6)

#### Psychometric properties of measures

Internal consistency estimates (Cronbach's Alpha) were satisfactory for all measures except for the K6, ranging from *α* = 0.55 to 0.77. Internal consistency estimates were as follows: K6 *α* = 0.55; WHODAS 2.0 *α* = 0.69; PHQ-9 *α* = 0.75; WHO-5 *α* = 0.77; AAQ-II *α* = 0.76.

Correlations between outcome measures are shown in Table 2 as an indication of convergent and discriminant validity. All correlations were in the expected directions, although they did not always reach statistical significance in this small sample. For example, psychological distress and depression showed positive correlations with functional impairment. Similarly, psychological flexibility was negatively correlated with psychological distress, functional impairment, and depression, and positively correlated with wellbeing, such that higher psychological flexibility was related to better outcomes, as expected.

**Table 2.** Correlations between measures

	Distress (K6)	Functional Impairment (WHO-DAS)	Depression (PHQ9)	Wellbeing (WHO5)	Psychological Flexibility (AAQ-II)
Distress (K6)	1				
Functional Impairment (WHODAS)	0.31**	1			
Depression (PHQ9)	0.26*	0.39***	1		
Wellbeing (WHO5)	−0.20	−0.20	−0.53***	1	
Psychological Flexibility (AAQ-II)	−0.39***	−0.41***	−0.50***	0.33**	1

*p < 0.05, **p < 0.01, ***p < 0.001.

#### Attendance and changes over time

Attendance in the female group was high: 76% of women (*n* = 25) missed none or one (out of five) sessions, and 25% (*n* = 8) missed two or three sessions. Attendance in the male group was mixed: 38% of men (*n* = 12) missed none or one (out of five) sessions, 31% (*n* = 10) missed two or three sessions, and 31% (*n* = 10) missed four sessions.

We were unable to conduct post-intervention interviews with 11 participants [17%: three female (9%), eight male (25%)]. Pre- to post-assessment changes were in the expected direction on all measures and reached statistical significance despite the low sample size. We report findings from the overall sample below. Gender disaggregated findings can be found in Table 3. The K6 scores decreased 58% from a mean of 14.6 (s.d. = 4.1) to 6.1 (s.d. = 3.7). Functional impairment, as measured by WHODAS scores, decreased by 33% from an average of 27.4 (s.d. = 6.6) to an average of 18.4 (7.4). PHQ-9 scores decreased from 13.4 (5.1) to 4.2 (4.4), reflecting an average improvement of 69% in depression. The WHO-5 scores increased from an average of 32 (s.d. = 22.2) to 55.9 (21.5), an improvement of 75% in wellbeing. Psychological flexibility (AAQ-II) increased 75% from 22.5 (9.6) to 39.3 (8.7).

**Table 3.** Comparison of pre- and post-assessment measures (gender disaggregated)

Outcome	Gender	Pre-assessment, Mean (s.d.)	Post-assessment, Mean (s.d.)	Mean difference, Mean (s.d.)	Percent change (%)	95% CI for Mean difference	*t*	df	*p*
Distress (K6)	Female	15.62 (3.80)	6.07 (4.22)	−9.55 (5.90)	−61.14	−11.79 to −7.31	−8.73	28	<0.001
	Male	13.33 (4.19)	6.21 (3.11)	−7.13 (5.46)	−53.41	−9.43 to −4.82	−6.40	23	<0.001
Functional Impairment (WHODAS)	Female	28.07 (6.40)	18.28 (8.98)	−9.79 (10.24)	−34.88	−13.69 to −5.90	−5.15	28	<0.001
	Male	26.50 (6.78)	18.50 (5.12)	−8.00 (6.66)	−30.19	−10.81 to 5.19	−5.89	23	<0.001
Depression (PHQ9)	Female	15.72 (4.52)	3.59 (4.25)	−12.14 (6.15)	−77.16	−14.48 to −9.80	−10.64	28	<0.001
	Male	10.67 (4.45)	5.00 (4.46)	−5.67 (4.73)	−53.14	−7.67 to −3.67	−5.86	23	<0.001
Wellbeing (WHO-5)	Female	21.79 (18.72)	57.24 (21.65)	35.45 (26.14)	162.69	25.51–45.39	7.30	28	<0.001
	Male	44.33 (19.84)	54.33 (21.71)	10 (19.52)	22.56	1.76–18.24	2.51	23	<0.01

Outcome	Gender	Pre-assessment, Mean (s.d.)	Post-assessment, Mean (s.d.)	Mean difference, Mean (s.d.)	Percent change (%)	95% CI for Mean difference	*z*	df	*p*
Psychological Flexibility (AAQ-II)^a^	Female	18.97 (8.53)	44.34 (6.11)	25.38 (11.81)	133.74	20.88–29.87	4.70	–	<0.001
	Male	26.71 (9.31)	33.21 (7.49)	6.50 (8.83)	24.34	2.77–10.23	3.03	–	<0.01

a The Wilcoxon signed-rank test was used for the AAQ-II.

#### Intervention implementation

All groups were successfully conducted by the facilitator team. Across the 10 SH+ sessions conducted, there were many intervention adaptations suggested by facilitators in the session feedback forms, but fewer than five documented instances where a participant requested additional help or support from a facilitator (e.g. school fees, referral to other organizations), or where reports were suggestive of needing increased support or intervention from the more experienced social worker. All cases were adequately managed by the facilitator team using a manualized protocol that helped participants identify sources of support in their community (e.g. friends, village leaders) with some limited advice and supervision from the social worker and Geneva-based clinical supervisor (KC). As a result, supervision Skype calls from Geneva occurred less than once a week and was less than 1 hour in length.

### Phase 4: Process evaluation

We conducted semi-structured interviews with 18 participants (eight completers, 10 non-completers), four SH+ facilitators, and the supervising social worker. Detailed quotes highlighting themes that emerged from the qualitative data analysis are presented in online Supplementary Table S4. Sub-themes on the overall helpfulness of the program, appropriateness, involvement of family and friends, and proposed adaptations were described by all participants, though in more depth by completers. The theme of attendance was discussed primarily in interviews with participants who dropped out part way through the program. The facilitator and social worker perspectives were captured through the themes of training and supervision, barriers and facilitators to successful facilitation, the most useful intervention components, and integration.

#### Benefits

Overall, participants reported that SH+ helped reduce overthinking and stress. A number of participants also reported focusing their problem-solving on things that could be changed, as well as improvements in their awareness, sleep, and the ability to enjoy daily activities. For example, a participant said:

*This program helped me because before it started I had too much stress. I am alone with my children. Despite what I am going through, no one can provide a helping hand. Most of my relatives died during the war, no one cares about me. This program helped me reduce my stress and my mind is free because I picked up the most important piece of advice they gave me: they said to change what you can and leave what you cannot change. I have left all those bad things I used to think about.* (Female participant, 38 years)

Participants also indicated that the skills taught by the program helped reduce ethnic tensions and increased socially supportive behaviors. A female facilitator explained:

“*They said that women who are attending the program are changing because usually Dinka and Nuer do not talk or greet each other but those who go for the program talk and greet one another.”* (Female intervention facilitator)

In addition, moving towards values was a theme identified by facilitators as illustrated by the following quote:

“*It has also rewarded them in helping each other in situations of being kind to one's self and being kind to others, moving towards one's values.*” (Female intervention facilitator, 27 years)

The majority of participants considered the SH+ materials such as the illustrated manual and the worksheets useful. These participants indicated that they could easily understand and relate to the illustrations and several indicated that they had shared the illustrated manual with others in their community. Even those who were unable to read Juba Arabic indicated that they understood the meaning from the illustrations in the manual and enjoyed looking at the illustrations, particularly after attending the SH+ sessions.

#### Challenges and barriers

The fact that no basic goods or services were provided as part of SH+ delivery was one of the primary challenges that were mentioned by all participants, although the extent to which this was a barrier to attendance varied. Although the nature of the program, including the lack of material goods and services provided, was explained through the consent process and reiterated at various points of interaction with the research team and during the intervention, some participants experienced disappointment when they attended sessions and were not provided with material goods.

Problems with understanding the audio-recording, illustrated manual, and work-sheets were largely related to language. A minority of key informants reported that the Juba Arabic used in the recordings was difficult to understand. Others indicated that they would be more comfortable listening to a recording in their ethnic language. One participant who did not complete all five sessions said,

“*This program could be made better if the materials were written using the different languages. As you know, people here are from different ethnic tribes and if materials were written using those languages then reading would be easy*”. (Female participant, 38 years)

When confusion arose during a session, facilitators would pause the recording and offer a clarifying explanation.

As noted, literacy was mentioned as a barrier to understanding the text in the illustrated manual and the worksheets. The issue of literacy was reported primarily for female participants, many of whom reported innovative ways to get around this obstacle. For example, an intervention facilitator said:

“*I think that mostly things were clear, except for the women's group, many of them were unable to read and write. So they would take their books to their children or neighbors to read it for them because a good number of them were unable to read. But for the men, they were able to read and write.*” (Female intervention facilitator)

The text-heavy nature of the worksheets that were part of the pilot version of SH+ was therefore considered a significant challenge for many participants not only due to illiteracy but also due to an inability to read Juba Arabic in some cases, which is used more commonly as a spoken rather than a written language.

Many participants cited competing priorities as a barrier to attendance. For example, some participants indicated that it was difficult to make time for the sessions given household and childcare responsibilities. Others indicated that attending every session was challenging given the many other activities ongoing in the camp. For instance, one of the SH+ sessions coincided with a food distribution activity that had been scheduled by another agency working in the camp making it challenging for participants to attend that day.

Finally, some male participants did not engage with the program indicating that some other men were talking when the intervention was ongoing and that there were men drinking alcohol before attending sessions, as illustrated by the following quote:

“*The thing that made me not attend the teaching those days was the issue that people talk, the ones who are there, and some people might first drink something and then they go and disturb people. They talk about different things when the facilitators are speaking.*’ (Male non-completer, 30 years)”.

Participants indicated that some younger male participants, in particular, did not find the program interesting and were not open to learning in that setting.

## Discussion

We applied a phased approach to translate, adapt, and pilot a guided self-help intervention with South Sudanese refugees in Uganda. We conducted group cognitive interviews with translated intervention materials; recorded suggestions for adaptations during training of facilitators and delivery of the groups; monitored changes on outcome measures before and after intervention in a first pilot implementation; and conducted a qualitative process evaluation.

Based on our study's results, we feel three key conclusions can be drawn from our findings. First, the applied adaptation methods resulted in both minor and more substantive adaptations at different stages and showed the importance of an adaptation process. Numerous minor changes to language and images were suggested during the cognitive interviewing, and the process evaluation highlighted the importance of scheduling intervention sessions so they do not clash with food distributions, vaccination campaigns, and other settlement events. Scheduling can be improved by closer coordination with other humanitarian agencies and consultation with the community advisory board regarding optimal timing. In addition, the training and pilot implementation identified a need to remove worksheets due to literacy issues, as well as a need to increase the role of facilitators (e.g. by introducing sessions, providing the instructions for group discussions instead of using the audio to do so, and answering questions to clarify content). The dilemma presented by the latter – that primarily relying on pre-recorded materials was considered a critical advantage of the SH+ format with regard to fidelity and scalability – was addressed by revising the facilitator manual to include clearer protocols to ensure the division between providing necessary information and guidance through the audio, while maintaining manualized, brief clarifications through facilitators. This approach retained the advantages of using audio-recorded materials and an illustrated manual over conventional group psychotherapeutic interventions (i.e. easier to ensure fidelity to intervention content, ability to have larger groups, reduced training, and supervision requirements).

With regard to major changes, we decided to focus further evaluation efforts through a randomized controlled trial with refugee women only based on the pilot implementation and process evaluation. We felt the low attendance of men, negative dynamics due to alcohol use by some participants, and observed disinterest of young men in particular, indicated the need for further intervention development with this population. Previous evaluations of group psychotherapy have found better impacts with women in this geographic region (Bolton *et al.*, 2003; Bolton *et al.*, 2007), so this remains a critical area for future investigation. Overall, the adaptations identified in this study are similar to adaptations reported in the mental health intervention literature in LMIC. A systematic review of adaptation efforts of depression interventions found that the most common adaptations are in the categories of language (e.g. use of colloquial expressions); therapist (e.g. adjusting therapeutic style); contextual fit (e.g., inclusion of family members if requested); and methods (e.g. reducing tasks relying on literacy) (Chowdhary *et al.*, 2014).

Overall, we feel the study findings highlight the importance, particularly with new or innovatively delivered interventions, of taking an iterative and comprehensive approach to adaptation that uses multiple methods drawing from diverse fields and views adaptation as a continuous process of improvement. Our experience of this approach has been that it resulted in a more feasible and easy to deliver intervention than would have been the case if the adaptation was viewed as a singular event occurring before piloting.

Second, we identified two challenges that may not be so easily addressed. A common complaint concerned the lack of provision of material support during the intervention. Since the process evaluation indicated that the majority of participants appreciated a focus on stress management, if possible, further clarifying the limitations of the intervention may be beneficial, though requests for meeting basic needs may still be expected given the extreme stressors faced by participants. In response, future applications of SH+ could consider integrating the intervention into livelihoods interventions if the evaluation shows the intervention to be efficacious. This may be a particularly interesting avenue because the format of the intervention (e.g. large group, relatively light training and supervision requirements) would allow for easier integration as compared with other psychotherapeutic interventions. If proven effective, SH+ could be more feasibly integrated into other humanitarian programs, which may assist in breaking observed vicious cycles between social determinants (e.g., poverty and intimate partner violence) and psychological distress (Lund *et al.*, 2011; Tol *et al.*, 2017).

Further challenges related to the use of Juba Arabic for the audio-recorded materials frequently arose. A choice for Juba Arabic was initially made in consultation with the community advisory board. Comprehension of intervention materials for some participants was challenging due to the different ways in which Juba Arabic is used by different groups in South Sudan. Future efforts could focus on developing intervention materials for the different languages spoken by large sections of the community in South Sudan, giving a choice of groups to be attended, and possibly recruiting community members to act as interpreters. However, in addition to time and logistical constraints, the advantage of increased comprehension of materials in ethnic languages needs to be weighed against the risk of showing partiality towards specific ethnic groups as well as the benefits noted in the process evaluation with regard to the increased positive social interaction between refugees of different ethnicities.

Third, an important finding is that the uncontrolled pilot showed promising results. Overall, findings regarding feasibility and acceptability of the research and intervention protocols were favorable. Psychometric properties of measures overall were satisfactory in terms of internal consistency, sensitivity to change, and convergent/ discriminant validity. Moreover, comparison of pre- and post-intervention scores for both sexes were in the expected direction for all outcome measures. These findings provide scope for future more rigorous evaluation in this context, which is critical to identify whether this innovative adapted intervention reduces psychological distress.

These conclusions need to be read in light of the following limitations. First, the group cognitive interviews with laypersons did not lead to substantial suggestions for adaptations, in contrast to the more detailed suggestions from group cognitive interviews with health workers, as well as the comments from intervention participants in the process evaluation. A recommendation for future use of group cognitive interviewing on multi-media materials is to listen to the recorded audio in a mock group format instead of reading through a script in order to increase validity and attention. Second, the pilot study's assessment of pre- and post-intervention scores was completed with a small convenience sample without a control group, and did not take into account participants lost to follow-up (17%: three women, eight men). These results should not be interpreted as an indication of the effectiveness of the intervention but rather as an indication of the potential appropriateness of the assessment measures and trends that should be explored further in future controlled studies. Third, the analysis of the qualitative process evaluation data was conducted by one person, increasing the risk for subjectivity in analyses.

Notwithstanding these limitations, our findings are promising and indicate that delivery of an innovative, scalable psychological self-help intervention to large groups of participants in challenging settings is feasible with minimal training and supervision, and was well-received by female participants. As such, the research contributes to a wider consideration of opportunities and challenges associated with the global dissemination of psychological interventions (Fairburn & Patel, 2014; White *et al.*, 2017). We have planned a small cluster randomized cluster trial to test the feasibility of research procedures, and a fully powered randomized controlled trial as a more rigorous evaluation of the potential benefits of SH+ in South Sudanese women. A further adaptation for use with men is warranted in the future.

## Supplementary material

For supplementary material accompanying this paper visit http://dx.doi.org/10.1017/gmh.2018.14. click here to view supplementary material
