# Supplementary material for: Translation, adaptation, and pilot of a guided self-help intervention to reduce psychological distress in South Sudanese refugees in Uganda
Source: Glob Ment Health (Camb). 2018 Jul 27;5:e25. doi: 10.1017/gmh.2018.14 (PMC6094403; doi:10.1017/gmh.2018.14)
Supplement: Supplementary file 1 [file S2054425118000146sup001.docx]

Table 4. Process evaluation themes by category of key-informant

| Theme | Sub-themes | Example |
| --- | --- | --- |
| Overall helpfulness | Reduces bad thoughts and stress | “The good thing I got there were the exercises and every time on my way home I would meditate. When I reach home, I feel very happy. For sure on this earth there are a lot of bad thoughts in our heads. No soap, no nothing, and if one puts her heart on thoughts, a person can die of too much thinking.” (Female participant, age) |
|  | Relaxes the body and the mind | “The exercises I learnt help me every day because each time I become tired, I do what the grounding exercise told us to do during the workshop and it always relaxes the body and the mind.” (Female non-completer, 33 years) |
|  | Improves relationships and reduces conflict | “Because of this program my daily life has changed. I stay with friends and neighbors and I am able to talk to them if there is any problem.” (Female participant, 38 years) |
|  | Problem-solving skills | “This program reduced my stress since it taught us that in the case where one cannot change a situation or condition, we should leave it and focus on what we can manage.” (Female participant, 38 years) |
|  | Improves sleep | “I used to take a few hours for sleeping. The rest of the hours I would think a lot, but after the program at least I have enough rest because I have eliminated all of the hard thoughts.” (Male participant, 49 years) |
| Adherence | Problem with understanding materials | “You see my daughter, I cannot even read the things that have been written in Arabic. You should also try to cater for those who cannot read Arabic like Dinka, Nuer, or Kakwa so that those who fail to read Arabic can understand also what has been written in the books.” (Female non-completer, age) |
|  | Competing responsibilities and length of sessions | “I saw an issue of time. People would go late and there would be work at home and then one would think I want to hurry back for the work. This is the thing that is difficult.” (Male non-completer, 30 years) |
|  | Disruptions and negative group dynamic | “The thing that made me not attend the teaching those days was the issue that people talk, the ones who are there, and some people might first drink something and then they go and disturb people. They talk about different things when the facilitators are speaking.” (Male non-completer, 30 years) |
| Appropriate-ness | Not appropriate for severely affected individuals | “On the other side, it might not help because there are people whose relatives were killed and they watched it happen. For such a person, he or she can become crazy and it is very hard to forget that scenario unless they are taken to a hospital for treatment or counselling.” (Female non-completer, 33 years) |
| Proposed adaptations | Adapt book with more pictures and fewer words | “The pictures are not enough for me and I would prefer more of the pictures than words written in the book.” (Female non-completer, 33 years) |
|  | Sensitize potential participants ahead of time | “In the beginning people had negative attitudes toward this program. Many of us were registered and some decided to leave the sessions, but I went as far as the end. I have learnt a lot from it. So I guess in another village, I think the first thing is to sensitize the community so that they understand the importance of the program. Then you will succeed.” (Male participant, 64 years) |
|  | Expand the program and provide materials to everyone who is distressed | “There are many people here who are distressed and I ask you people to at least try and give out the materials to every person who wants to be reading in times of distress. The books have pictures and can help some people relieve their stress just by looking at them.” (Female non-completer, age) |
|  | Allowing facilitators more autonomy | “They were limited and they could not interact with the participants also the participants could not really interact with the participants so it was really a challenge to me.” (Social worker) |
| Involvement of family and friends | Sharing materials with others | “Yes, I gave [the illustrated guide] to the person whose house collapsed. I told him to read it. He read it and said the things are okay and he understood. My book is still with him even.” (Female participant, 40 years) |
|  | Practicing exercises with others | “If we sit in one place with other people, I tell them the exercises that we learnt. Even during the day like this I sit with my children and tell them to do the exercises that we learnt.” (Female participant, age) |
| Training and supervision | Examples and practice for trainees | “At first, it was not easy to understand anything at all even though I was reading the SH+ book and the worksheet. I didn’t understand until we started doing it practically through the various exercises.” (Female intervention facilitator, 27 years) |
| Barriers and facilitators to successful facilitation | Opportunity for participant feedback | “What I would change is that I would want to involve them in the aspect of the discussions or sharing the participant view. I would also get their views on how they understand the whole thing and their experiences in doing the exercises.” (Female intervention facilitator, 33 years) |
|  | Materials were not cross-referenced | “There is a need to number the worksheet in order to match with the audio so that it does not look as complex.” (Female intervention facilitator, 24 years) |
|  | Difficult to handle constant requests for material goods | “From our point of view, we told them openly that the exercise does not require material things. We are not going to offer them material gain or money, ours is the Self-Help Plus. All in all, they still continued demanding other materials, so that made it a bit challenging.” (Female intervention facilitator, 35 years) |
|  | Group format allowed participants to encourage one another to attend | “And also in the men’s group, one person took the initiative of mobilizing, though he was not told to do that, he took the initiative of trying to mobilize his friends to come to the group.” (Female intervention facilitator, 35 years) |
|  | Disruptions including use of alcohol | “The most challenging thing was that some of the people in my group, let’s say around five, were smelling of alcohol and one was drunk, so he was actually trying to interrupt the session.” (Female intervention facilitator, 35 years) |
|  | Time management | “Because it’s like there were few minutes given like five minutes for the break when snacks were provided yet they were supposed to go for calls so this five minutes is not enough so like when it come for time for the audio to start some of them were still with their sacks so this one interrupted their attention so there is need to improve on that time management.” (Social worker, 31 years) |
| Most helpful intervention components | Grounding and unhooking | “I mean participants have appreciated it and they now have the skills of managing stress. If you are stressed, you know how to ground yourself at your own home and how to unhook yourself.” (Female intervention facilitator, 45 years) |
|  | Befriending | “The program helps me every day. Before the program was brought, my daily life at home was not good with my children because of what is stressing me and I always finished my stress on them, but the program helped me a lot in handling such situations. I learnt that what I am not able to change, I should leave it and deal with what I am in position to change, as well as staying well with my neighbours.” (Female non-completer, 34 years) |
|  | Moving toward your values | “It has also rewarded them in helping each other in situations of being kind to one’s self and being kind to others, moving towards one’s values.” (Female intervention facilitator, 27 years) |
| Integration | Integrated care | “For us to achieve our goal there is a need to work hand in hand with others so that people are supported by all other services. The support may not necessarily be psychosocial support, the support may not be for mental problems, but other support such as financial, social support, spiritual support, so that we see that there is complete delivery of service to the community... and with this Self-Help Plus will work.” (Female social worker, 31 years) |
